# Views on Augmented Reality, Virtual Reality, and 3D Printing in Modern Medicine and Education: A Qualitative Exploration of Expert Opinion

**DOI:** 10.1007/s10278-023-00833-w

**Published:** 2023-05-10

**Authors:** Julie Urlings, Guido de Jong, Thomas Maal, Dylan Henssen

**Affiliations:** 1 Department of Neurosurgery, Radboud University Medical Centre, Geert Grooteplein Zuid 10, 6525 GA Nijmegen, The Netherlands; 2 3D Lab Radboudumc, Radboud University Medical Centre, Geert Grooteplein-Zuid 10, 6525 GA Nijmegen, The Netherlands; 3 Department of Medical Imaging, Radboud University Medical Centre, Geert Grooteplein 10, 6525 GA Nijmegen, The Netherlands

**Keywords:** 3D technology, Augmented reality, Virtual reality, 3D printing, Qualitative research

## Abstract

Although an increased usage and development of 3D technologies is observed in healthcare over the last decades, full integration of these technologies remains challenging. The goal of this project is to qualitatively explore challenges, pearls, and pitfalls of AR/VR/3D printing applications usage in the medical field of a university medical center. Two rounds of face-to-face interviews were conducted using a semi-structured protocol. First an explorative round was held, interviewing medical specialists (8), PhD students (7), 3D technology specialists (5), and university teachers (3). In the second round, twenty employees in high executive functions of relevant departments were interviewed on seven statements that resulted from the first interviewing round. Data analysis was performed using direct content analyses. The first interviewing round resulted in challenges and opportunities in 3D technology usage that were grouped in 5 themes: aims of using AR/VR/3D printing (1), data acquisition (2), data management plans (3), software packages and segmentation tools (4), and output data and reaching end-user (5). The second interviewing round resulted in an overview of ideas and insights on centralization of knowledge, improving implementation of 3D technology in daily healthcare, reimbursement of 3D technologies, recommendations for further studies, and requirement of using certified software. An overview of challenges and opportunities of 3D technologies in healthcare was provided. Well-designed studies on clinical effectiveness, implementation and cost-effectiveness are warranted for further implementation into the clinical setting.

## Introduction

Since the early 1990s, innovative visualization techniques including augmented reality (AR) and virtual reality (VR) have been slowly but progressively introduced in modern medicine. Popular applications focused on visualizing complex anatomical structures and combine these online models with virtual training and operation planning features. For a review on AR/VR in anatomy education, see [1]; for exemplary reviews on AR/VR in surgeon’s training, see [2–5]. The domain of AR/VR has been extended to encompass medical educational purposes, communication facilitation, and a wide range of therapeutic interventions [4, 6–12]. Approximately 12,000 scientific publications were retrieved when searching for VR applications in medicine [13]. Furthermore, the global market for VR and AR applications in healthcare is expected to reach $5.1 billion USD by 2025 [14]. In addition, developments in AR and VR are expected to propel 3D printing in medicine [15]. Together with AR and VR, 3D printing is believed to have great potential to transform how physicians access and view medical imaging data [16]. Furthermore, research has shown that AR, VR, and 3D printing could improve patient education and help to create well-informed patients suffering from various diseases [17, 18], potentially improving therapeutic compliance [19, 20].

Despite the rapid developments and the promising results of AR/VR/3D printing in research over many decades, full integration of these new techniques remains unaccomplished; AR/VR/3D printing is still not standard practice and is not regularly used in daily healthcare. This article qualitatively investigates the challenges, pearls, and pitfalls of AR/VR/3D printing applications in the medical field. Considering both the achievements and the remaining challenges, this study aims to identify trends towards possible future developments.

## Materials and Methods

### Ethical Approval and Participant Recruitment

This study was conducted at the Radboud University Medical Center in Nijmegen, the Netherlands. After consultation of the local ethical review board, ethical approval for this study was waived. To recruit participants, various online advertisements were placed with a 2-week interval. Also, to ensure that all eligible participants were identified, two recruiting e-mails were sent to all chairs of the departments of our university medical center. Between these two e-mails, there was an interval of 4 weeks. In these recruiting advertisements and e-mails, the researchers explained the methodology and goal of this study. Two rounds of qualitative interviewing were conducted. No strict inclusion or exclusion criteria were defined by the researchers, allowing for anyone with an interest in AR/VR/3D printing to be interviewed.

### First Interviewing Round

**Fig. 1:**
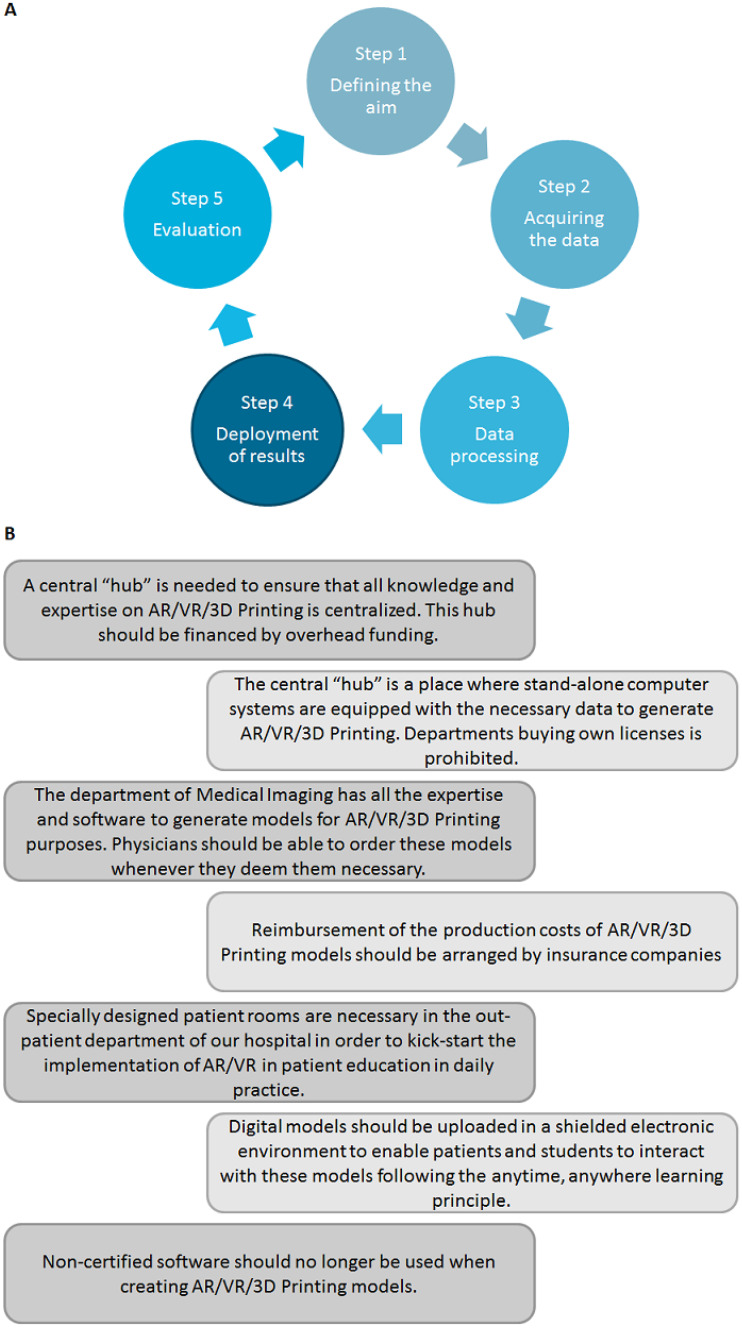
Structure of interviewing rounds

The first round of interviewing concerned an exploratory round in which residents, practicing medical doctors, PhD students, 3D technology software specialists (employees Radboudumc 3Dlab), and university faculty, both junior and senior, were interviewed to express their thoughts, wishes, expectations, and views on possible challenges. Eligible participants were contacted by e-mail containing more detailed information on the purpose of the interviews and the research methodology. Participation was voluntary. After agreeing to participate, an online interview setting was arranged by using Skype. Audio-recorded informed consent to be interviewed and recorded was obtained from all participants prior to the start of the interview. Semi-structured interviews were conducted to obtain detailed descriptions and extensive data of the participants’ perspectives on AR/VR/3D printing applications. The interview schedule of the first round was structured by using topics suggested by discussion between three investigators. These topics were enriched by seemingly important topics derived from the literature [21]. Interviews were structured by using the first five steps in the data lifecycle management cycle as proposed by the cross industry standard process for data mining (CRISP-DM) model: (1) defining aim; (2) acquiring the data; (3) data processing; (4) development of results; and (5) evaluation (Fig. 1A) [21]. This first round of interviews was conducted by one of the researchers in collaboration with one of the student-investigators under the supervision of one of the researchers who is experienced with regard to qualitative research methods. All interviews were audio-recorded. All recorded interviews from the first interviewing round were transcribed semi-verbatim.

The data from the interviews were independently analyzed using direct content analysis by two researchers [22]. This was done using Atlas.ti software version 22 Windows (http://atlasti.com; ATLAS.ti Scientific Software Development GmbH, Berlin, Germany). The constant comparative method was carried out after finishing the first interview following an inductive iterative process. This means that data analysis started after the first interview was completed. Codes derived from the previous interview were used as a starting point for coding the next one, adding additional codes whenever needed. This process was carried out until saturation was achieved, i.e., when no new codes arrived during an interview. Thereafter, two additional interviews were held.

Axial coding, in which codes were linked together and combined into categories, was also started and was continued throughout the first interviewing round. Out of the identified categories themes were derived.

### Second Interviewing Round

The second round of interviewing was based on the recommendations and outcomes of the first round of interviewing. By recommendation, the chairs of departments with relevant experiences with AR, VR, and/or 3D printing were contacted in which the goals and aims of this study were explained. Chairs were asked to recommend specific employees in high executive functions (including themselves) with a vision of the future of AR, VR, and/or 3D printing to take part. For this round, executives were chosen because they are the ones deciding whether or not and in what way to implant 3D technologies in daily healthcare.

Reminding e-mails were sent up to three times after the initial e-mail. All interviews in the second round of interviewing were audio-recorded, and again, audio-recorded informed consent was obtained from all subjects. Based on the codes and themes resulting from the first round of interviewing, seven statements on the implementation and centralization of 3D technologies in a healthcare setting were extracted by two researchers. These statements can be viewed in Fig. 1B “Structure of interviewing rounds”. Interviews were structured by the discussion of these seven statements. All recorded interviews from the second interviewing round were transcribed verbatim.

Data analysis was identical to qualitative data analysis for the first round of interviewing as described above (see “First Interviewing Round” section). All coding was performed using Atlas.ti software V.8.4.2 (http://atlasti.com; ATLAS.ti Scientific Software Development GmbH, Berlin, Germany) by two investigators.

## Results

Twenty-three participants, including residents, practicing medical doctors, PhD students, 3D technology software specialists, and university faculty, were interviewed during the first round of interviewing. Twenty participants were interviewed during the second round of interviewing. These employees in high executive office were appointed to several departments (anatomy, cardiology, dentistry, general surgery, neurosurgery, information management, medical imaging, oral and maxillofacial surgery, orthopedics, otorhino­laryngology, plastic surgery, rehabilitation medicine, the 3D lab, health academy, health innovations lab). Details can be viewed in Table 1. Interviews lasted between 20 and 65 min.

**Table 1:** Participant characteristics

**Round 1**	**Participants (23)**
**Role**	
- **Residents**	3
- **Practicing medical doctors**	5
- **PhD students**	7
- **3D technology software specialists**	5
- **University faculty**	3
**Round 2**	**Participants (20)**
- **Anatomy**	1
- **Cardiology**	1
- **Dentistry**	1
**- General surgery**	2
- **Neurosurgery**	1
- **Information management**	1
- **Medical imaging**	2
- **Oral and maxillofacial surgery**	2
- **Orthopedics**	2
- **Otorhino­laryngology**	1
- **Plastic surgery**	1
- **Rehabilitation medicine**	1
- **The 3D lab**	2
- **Health academy**	1
- **Health innovations lab**	1

### First Round of Interviewing

#### The Aims of Using AR/VR/3D Printing

Interviewees expressed that their goals regarding AR/VR/3D printing were threefold. With regard to educational purposes, it was reported that residents used either technique to learn specific skills, especially regarding laparoscopic surgery (For an overview of the derived themes and categories of round 1 see Table 2). By the use of this virtual environment, a safe learning space was created where residents could practice their skills on a voluntary basis. Next to a virtual learning environment, this facility also caused that the interviewed residents felt more secure about their own skills. They also reported to feel more in control about their own learning process. In addition, due to the trial-and-error situation without consequences, residents and their supervisors were more aware of the capabilities of the resident, which enabled supervisors to supervise their actions in a more tailor-made fashion. Regarding educational aspects for medical students, university faculty expressed that the use of AR and VR models enhanced anatomy education as it could help students to study following an anytime anywhere principle. Also, students were more in control with regard to creating their own perspective on an anatomical structure, especially on structures that would otherwise be difficult to visualize or interact with (e.g., especially cavities such as the brain ventricles or the pterygopalatine fossa).

**Table 2:** Themes and categories round 1

**Themes**	**Categories**
**The aims of using AR/VR/3D printing**	- Educational purposes - Research - Goals in healthcare
**Acquiring data to create models suitable for AR/VR/3D printing**	- Radiological data - 3D camera
**Data management plans for AR/VR/3D printing**	- Requirements - Consulting privacy officers - Safe data transfer
**Software packages and segmentation tools**	- Freeware applications - In-house written software - Clinically approved software packages - Difficulties of software packages
**Obtained output data and results reach the end-user**	- Obtained output data - End-users: scientist or practicing medical doctors - End-users: patients, students, and residents

In the research setting, validation of certain imaging techniques was described as the major aim of using AR/VR/3D printing. Two illustrative examples were given. With regard to 3D printing, when designing a mandibular prothesis, this design needs to mimic the configuration of the resected mandible, though it also needs to function as the resected bone did. Another example concerns the implementation of AR/VR in the educational setting. Studies of the impact on student motivation, study results, and other outcomes need to be carried out in order to validate whether these “nice to have” features are also valuable. Therefore, validation of AR/VR/3D printing in the academic setting was the prime focus of research regarding these techniques.

With regard to the goals of healthcare, it was believed that 3D models in the broadest term would greatly enhance medicine of the future as all 3D models (when based on radiological data) could provide insight in radiomics of tissue. When aligned with artificial intelligence, these developments could predict outcomes which are directly relevant for patients. More particularly, patient education by the use of AR/VR/3D printing was discussed as an important goal. It was expressed that these new techniques were believed to enhance patient understanding of a specific disease or treatment. Besides, when comprehension was increased, interviewees expressed that this could improve therapy compliance. Including patients in this process could help to construct patient-friendly, useful, and more effective AR and/or VR applications. However, it was also expressed that AR/VR/3D printing was probably not useful in every disease; some disorders were deemed more appropriate than others. More elusive and complex diseases, as well as those conditions not visible from the external surface (e.g., cerebral tumors, aneurysms), were believed to benefit more from the combination of standard patient consultation and AR/VR/3D printing. Finally, it was reported that AR/VR/3D printing could help patients to explain their disease to their relatives and improve awareness and understanding.

#### Acquiring Data to Create Models Suitable for AR/VR/3D Printing

In general, interviewees used serial radiological data (computed tomography (CT) and magnetic resonance imaging (MRI) scans). In some cases, a 3D camera system, sometimes equipped with infrared light, was used. Radiological data was obtained as DICOM files, which is the standard data file for these types of images. To further process the data, the DICOM files were converted to NIfTI files. Data concerned both in vivo and ex vivo data.

#### Data Management Plans for AR/VR/3D Printing

Data management plans needed to adhere to good clinical practice, ethical guidelines, and (inter)national jurisdiction. Privacy officers and data management specialists needed to be consulted according to most interviewees in order to ensure that data was stored safely and properly. Nevertheless, the most important factor for successful data management was the effectuation of a pipeline between the data storage space and the imaging visualization software and/or post-processing software.

#### Software Packages and Segmentation Tools

A diverse variety of freeware applications was used by the PhD students and the 3D technology software specialists (mostly ITK Snap (http://www.itksnap.org) and MeshLab (https://www.meshlab.net/)). In-house written software was also reported by the 3D technology software specialists to be used. Residents and university faculty used clinically approved software packages. All interviewees reported struggling at the beginning of their first AR/VR/3D printing project due to the vast number of possible options with regard to software and analysis techniques. In addition, highly protected computer systems within the university medical center environment did not allow for free installation of software. Only “approved” software could be installed, which complicated the pipeline from data storage spaces and the further processing of the retrieved data. In response to that, well-protected stand-alone devices are bought independently by different departments. On these stand-alone devices, the department downloads and installs the necessary software packages which are not allowed to be installed on computer systems which are part of the hospital network. Thereby, various interviewees reported to have paid for and installed identical software packages independently.

#### Obtained Output Data and Results Reach the End-User

The obtained output data comprises electronic 3D models (AR/VR) and/or the 3D-printed models. Depending on the purpose of these models, the output could differ. Electronic 3D models comprised StandardTessellationLanguage files (.stl-files) and Wavefront OBJ files (.obj-files).

Regarding the question whether the AR/VR/3D printing models reach the end-users, the interviewees were divided. When the end-user was defined as the scientist or practicing medical doctors, the interviewees expressed that these end-users were enabled to see and interact with the acquired models. However, regarding patients, students, and residents, substantial improvements could still be made. The AR/VR models could not be incorporated into the electronic patient file and thereby could not be interacted with by the patients. Furthermore, AR, VR, and 3D-printed models were primarily made in experimental settings. With regard to the sparsely created 3D-printed models, most models are distributed to the patients. However, to implement this on a larger scale, a fitting reimbursement needs to be effectuated to cover the costs of production.

When discussing student education, it was noted that the AR applications were finding their way towards the individuals’ devices to be used following the anytime and anywhere learning principle. The same, however, was not the case for the VR model. On the other hand, 3D models already form a cornerstone of anatomy education. The use of 3D printing to create models that are unavailable but are required according to the university faculty is being implemented progressively over time.

### Second Round of Interviewing

The seven statements, as shown in Fig. 1B, are discussed with all the interviewees to explore their vision and thoughts on the use of AR/VR/3D printing in education, research, and healthcare in a university medical center.

#### Centralizing All Knowledge and Expertise on AR, VR, and 3D Printing

When discussing centralizing all knowledge and expertise on AR, VR, and 3D printing, interviewees were in favor of this idea, but only if certain conditions would apply. The central hub would need to be a service rather than a selection committee that decides on which projects will be supported and which will not, especially if the hub itself is financed by overhead funding (For an overview of the statements and themes of round 2 see Table 3). Certainly, experts would review proposals critically and, if possible, improve proposals concerning AR/VR/3D printing. It was reported unfavorable that such a hub would carry out strict selection on which projects to support. Furthermore, as every employee of the university medical center would indirectly pay for its services if it were to be funded by overhead funding, one of the other conditions was that the hub would aim to support education, research, and health-related projects. The final condition for this proposal would be a broad support base for ideas and projects.

**Table 3:** Statements and themes round 2

**Statements**	**Themes**
**Centralizing all knowledge and expertise on AR, VR, and 3D printing**	- Central hub as a service - Central hub aims to support education, research, and health-related projects - Broad support base for ideas and projects
**Use of a physical central hub**	- Advantages of a physical central hub - Disadvantages of a physical central hub
**Improved implementation of the use of 3D models in daily healthcare**	- Easy to implement
**Scenarios concerning reimbursement**	- Insurance companies - Reimbursement by patients - Recommendations for future studies on the effectiveness of AR/VR/3D prints
**Specially designed consultation rooms in out-patient departments**	- Evidence is lacking - Qualification of medical professionals in teaching by the use of innovative models
**Electronic environments for end-users**	- Requirements of electronic environment - Viewing models on personal device
**Use of certified software**	- Certified software for clinical practices - More freedom for research and educational purposes

#### Use of a Physical Central Hub

The use of a physical central hub to place specially equipped hardware was met with controversy. At first, most interviewees agreed that such a physical space would be useful, but upon second thought, most interviewees saw more disadvantages than advantages. It was considered that the relatively small reduction in costs would not outweigh the disadvantages of diminished autonomous use and decreased accessibility of software.

#### Improved Implementation of the Use of 3D Models in Daily Healthcare

With regard to the improved implementation of the use of 3D models in daily healthcare, interviewees were enthusiastic. Such services would be relatively easy to implement and, in a way, are already present. For example, representatives of the departments of medical imaging, neurosurgery orthopedic surgery, oral and maxillofacial surgery, and trauma surgery reported that requests for 3D models of fractures or cerebral aneurysms are already created upon request.

#### Scenarios Concerning Reimbursement

The problem, however, concerns the reimbursement. With regard to the reimbursement of the time of the employee who creates the 3D model and, when it concerns a 3D-printed model, costs of the hardware (e.g., 3D printer) and materials used, several scenarios were heard. First, if research shows that the use of AR, VR, and/or 3D prints was considered a functional, evidence-based technique that improves patients’ understanding of their disease, it would be necessary that insurance companies reimburse the costs. Second, if research, however, reports these techniques to be an add-on modality that does not result in lower costs of the healthcare processes or improved patient satisfaction, outcome, or safety, it should not be reimbursed by insurance companies. More extremely, it was said that if research shows no evidence for the use of AR, VR, and/or 3D printing, it should be avoided. However, an alternative view on this latter scenario was that when patients wanted to have a 3D model of their illness even though research had not shown an additional value, the service could still be offered. The costs, however, should be reimbursed by the patients themselves. Concerning the use of AR/VR/3D prints in educational settings, program directors were enthusiastic to aid further integration of these techniques in (bio)medical curricula. When focusing further on recommendations for future studies to help investigate the effectiveness of AR/VR/3D prints, it was stated that future clinical studies should also focus on the implementation process of the interventions to increase the effectiveness of these applications in practice. Feasibility and adequacy in the clinical setting should be assessed to elucidate which method is favorable in each setting. Additionally, the costs of creating AR, VR, and 3D prints should also be investigated, and cost-effectiveness studies are needed to elucidate whether these costs outweigh the benefits.

#### Specially Designed Consultation Rooms in Out-Patient Departments

When discussing specially designed consultation rooms in the out-patient departments, all interviewees were critical. If it were found that these techniques could be useful, the interviewees stated that the necessary changes would have to be carried out. However, as long as evidence was lacking, such permanent changes were not considered to be necessary. However, three participants wondered whether medical professionals were equipped to teach their patients by the use of such innovative models. They proposed to design special educational programs for medical professionals on how to use such models effectively to optimize patient education by the use of AR, VR, and/or 3D prints.

#### Electronic Environments for End-Users

Furthermore, all interviewees believed that well-designed, well-protected electronic environments were needed to reach the end-users of these technologies, whether they were students or patients. In addition, five interviewees stated that when patients could use the models on their own devices, no special changes were needed in the consultation rooms of the out-patient departments.

#### Use of Certified Software

The final statement regarding the use of certified software was reported to be two-sided. All interviewees agreed that for clinical practices, especially when basing healthcare decisions on 3D models, the post-processing software legally needed to be tested and approved by the relevant institutions (e.g., Food and Drug Administration (FDA) approved; Conformité Européenne (CE) marked). For research or educational purposes, the interviewees were less strict. They admitted that in these settings, more freedom with regard to the creation of well-designed models would be preferable.

## Discussion

This paper provided an overview of the challenges and opportunities experienced by experienced users of AR/VR/3D prints in a university medical center in the Netherlands and used these insights to qualitatively investigate the views and opinions of employees in high executive functions with affinity to the topic to identify trends towards possible future developments. Regarding the educational and research purposes of AR/VR/3D prints, the interviewees expressed that these implementations were ongoing and extensively investigated. More specifically when focusing on the use of AR/VR/3D prints in educational settings, university faculty and program directors were enthusiastic about aiding further integration of these techniques in (bio)medical curricula. It was found that although the technological possibilities have rapidly developed over the course of 30 years, studies on clinical effectiveness, clinical implementation, and cost-effectiveness are lacking.

### Covariates Which Might Affect Effectiveness of Using AR/VR/3D Prints

Various papers reported improved knowledge retention in patients suffering from specific disorders [23–27]. Nevertheless, not every disorder is expected to benefit from patient education with AR/VR/3D printing. Furthermore, inter-individual differences and characteristics that significantly impact student performance when working with AR/VR/3D prints have not been studied when investigating the effectiveness of AR/VR/3D prints in medical education (for a recent review, see [28]). One of the most important factors concerns an individuals’ spatial insight. It is well-known from papers on the use of AR/VR/3D prints in anatomy education that differences in pre-intervention spatial ability confounded the learning results [29–32]. Whether spatial abilities and other co-variates play a significant role when working with AR/VR/3D prints in patient education remains elusive and should be investigated in future studies. Furthermore, whether AR, VR, or 3D prints are more effective in different patient populations still remains topic of debate.

### Cost-Effectiveness and Clinical Implementation

Another important understudied field of research concerns the cost-effectiveness of AR/VR/3D prints in medicine. This remains an important omission of the scientific literature [33]. It has been stated that improvements in cost-effectiveness, access, and usability of 3D printing technology have enabled surgeons to increasingly use this powerful tool [34]. However, to the authors’ knowledge, no cost-effectiveness analyses on the use of 3D Printing have been published. With regard to immersive VR as a training facility for residents, it was found that low-fidelity simulators were more cost-effective than high-fidelity simulators [35]. In addition, a more recent systematic review also concluded that virtual training could be considered as a cost-effective teaching tool for residents, although a true cost-effectiveness analysis is lacking [36]. As these analyses are still lacking, further clinical implementation is held back.

### Strengths and Limitations

One of the strengths of the current paper is the qualitative design and the use of two rounds of interviews to include experienced users of AR/VR/3D print technologies as well as professors and visionaries with affinity about this subject. One limitation concerns the method used to select subjects for the second round of interviews, as asking single individuals in leadership roles for recommendations could have led to selection bias. Another limitation of this study is the limited generalizability of the results and recommendations regarding specific institutions, as this study was conducted in only one university medical center in the Netherlands. Nevertheless, the reported recommendations can also be useful for other institutes to improve their infrastructure with regard to AR, VR, and/or 3D printing. Furthermore, the most important omissions of the field of AR, VR, and/or 3D prints have been identified. Along with the reported trends, some suggestions for possible future developments have been made to help accelerate the integration of AR/VR/3D prints into the daily practice of modern medicine. However, to increase the likelihood of successful and relevant implementation of these new techniques in modern medicine, the patient’s perspective should not be forgotten. Although beyond the scope of the current study, more research should be conducted regarding patients’ expectations of AR/VR/3D printing in modern medicine.

## Conclusions

Qualitative exploration has provided an overview of the challenges and opportunities of AR, VR, and/or 3D prints in modern medicine and education. Especially concerning clinical usability and implementation, further developments are warranted. For further implementation into the clinical setting, well-designed studies on the clinical effectiveness, clinical implementation, and cost-effectiveness are obligatory.

## Data Availability

The data that support the findings of this study are available from the corresponding author, JU, upon reasonable request.
